# What about the men? Perinatal experiences of men of color whose partners were at risk for preterm birth, a qualitative study

**DOI:** 10.1186/s12884-020-2785-6

**Published:** 2020-02-10

**Authors:** Brittany N. Edwards, Monica R. McLemore, Kimberly Baltzell, Allen Hodgkin, Olga Nunez, Linda S. Franck

**Affiliations:** 1 Central California Faculty Medical Group/UCSF Fresno, 2625 E Divisadero St, Fresno, CA 93721 USA; 2 University of California, San Francisco, USA; 3 Fresno Housing Authority, Fresno, USA; 4 California State University, Fresno, USA

**Keywords:** Men of color, Fathers, Fatherhood, Parental role, Preterm birth, Pregnancy, Neonatal intensive care unit, Discrimination, Patient-provider communication

## Abstract

**Background:**

Preterm birth in the United States is associated with maternal clinical factors such as diabetes, hypertension and social factors including race, ethnicity, and socioeconomic status. In California, 8.7% of all live births are preterm, with African American and Black families experiencing the greatest burden. The impact of paternal factors on birth outcomes has been studied, but little is known about the experience of men of color (MOC). The purpose of this study was to explore the experiences of MOC who are partners to women at medical and social risk for preterm birth.

**Methods:**

This study used a qualitative research design and focus group methods. The research was embedded within an existing study exploring experiences of women of color at risk for preterm birth conducted by the California Preterm Birth Initiative.

**Results:**

Twelve MOC participated in the study and among them had 9 preterm children. Four themes emerged from thematic analysis of men’s experiences: (1) “Being the Rock”: Providing comfort and security; (2) “It’s a blessing all the way around”: Keeping faith during uncertainty; (3) “Tell me EVERYTHING”: Unmet needs during pregnancy and delivery; (4) “Like a guinea pig”: Frustration with the healthcare system. Participants identified many barriers to having a healthy pregnancy and birth including inadequate support for decision making, differential treatment, and discrimination.

**Conclusions:**

This study shows novel and shared narratives regarding MOC experiences during pregnancy, birth, and postpartum periods. Healthcare providers have an essential role to acknowledge MOC, their experience of discrimination and mistrust, and to assess needs for support that can improve birth outcomes. As MOC and their families are at especially high social and medical risk for preterm birth, their voice and experience should be central in all future research on this topic.

## Background

Preterm Birth is defined as a baby born before 37 weeks of gestation [1]. According to the Centers for Disease Control and Prevention, almost 1 in every 10 live births is born premature [1]. Preterm birth is a major cause of infant mortality in the U.S. and annually creates over a $25 billion economic burden for society. Causes of preterm birth are not fully understood but are influenced both by clinical and social factors, including high blood pressure, diabetes, ethnicity, socioeconomic status, and chronic stress [2]. During 2014–2016, the total U.S. preterm birth rate increased by 4% [3]. Non-Hispanic Black women were most at risk for preterm birth with a rate of 13.6% [3]. Hispanic and Non-Hispanic White women had lower rates of 9.45 and 9.06% respectfully [3]. Although California has a lower national preterm birth rate overall at 8.7%, Black women still have 49% higher rate than all other women [4].

Research that involves listening to women of color (WOC) has shed new light on the relationships between racism and perinatal health outcomes. For example, one recent study found chronic worry about racial discrimination was associated with preterm birth rates in Black women with higher income [5]. Perceptions of racial discrimination were also associated with low-birth weight newborns [6]. Black and Hispanic/Latina women at risk for preterm birth have reported experiencing disrespect, inconsistent social support, stressful interactions during healthcare encounters, but also confidence with newborn care [7]. However, little is known about the experience of men of color (MOC) in the U.S. who are partners of at-risk women and fathers of preterm newborns.

Several international qualitative reviews have summarized the experience of fathers during pregnancy and birth. The reviews are largely based on studies with ethnically homogenous groups from high resource settings such as the United Kingdom, Australia, and Sweden [4, 8–14]. Investigators found that first-time fathers experienced a transition during pregnancy, from an initial apprehension to acceptance of the pregnancy [12]. Fathers in the studies commonly reported feeling distant from their pregnant partners, separated in a “mother-centered” health system, and needing more information to support their partner [12–14]. During the unexpected event of preterm birth, feelings of stress, fear, depression and shame dominated fathers’ experiences [9].

There are very few studies examining the experiences of MOC in the U.S., as the literature has been historically based on the experiences of White men [15]. One study reported perceptions of prenatal care for homeless women in an African American community in Washington D.C. [16]. Male partners (*N* = 39) reported being perceived as both a barrier and motivator for women receiving care. Another study described experiences of 18 adolescents who self-identified as Latino and mixed race, within juvenile systems who became fathers at a young age [17]. Most of the young men reported experiencing abusive relationships with their own fathers and traumatic life events, and yet they envisioned being supportive fathers to their own children. Consequently, there is insufficient evidence about pregnancy and birth experiences of MOC in the U.S.

Greater father involvement is associated with decreased rates of preterm birth and low birthweight rates [18]. However, higher trends of paternal absence continue to persist in communities of color in the U.S. [19] and for families with absent fathers, the risk of infant mortality may be four-times higher in Black families than White families [20]. Recent census data shows that 38% of African American minors live with both parents in comparison to 73% of White minors [21]. Researchers have speculated this may be largely due to systemic and institutional policies such as unjust policing systems or assistance programs providing perverse financial incentives to low-income mothers for living alone [22, 23]. Additionally, MOC are more likely than White men to live in poverty, reside in polluted environments, be exposed to toxic substances, experience violence, and work in dangerous occupations [24]. The accumulation of trauma and stress on MOC impacts not only themselves, but their families. For these reasons, it is crucial that research is conducted to learn about the perceptions and involvement of MOC in pregnancy and birth.

Historically, people of color (POC) have been subjects of unethical research and have appropriately developed medical mistrust of healthcare providers (HCPs) and investigators [25–27]. Research that begins with listening to MOC provides an opportunity for researchers and HCPs to not only rebuild trust and improve relationships, but better understand and care for those at highest risk for preterm birth.

This study aimed to explore the experiences of MOC who are partners to women at risk for preterm birth. The specific research questions for the study were: (1) what are the personal experiences of MOC during their female partner’s pregnancy, birth, and the first few months after birth? and (2) what do MOC perceive as barriers and facilitators to having a healthy pregnancy and newborn?

### Operational definitions

For purposes of this study, the following definitions were specified: Men of color (MOC), women of color (WOC), and people of color (POC) are defined as individuals in the U.S. who self-identify as African American or Black, Hispanic or Latinx, Asian, Indigenous, and other ethnicities other than White and/or European. Social (environmental) risk of preterm birth refers to social and environmental determinants that have been found to increase risk of preterm birth, such as racism and chronic stress [28]. Medical risk of preterm birth refers to maternal physiological risk factors such as previous preterm birth, short cervical length, and intrauterine infections during pregnancy [29].

## Methods

### Study design and setting

This qualitative study was nested within a larger project conducted through the California Preterm Birth Initiative (PTBi-CA). The PTBi-CA is a multi-year research collaboration striving to decrease the burden of preterm birth in Fresno, Oakland, and San Francisco, CA. In the larger study, the lead investigators conducted focus groups with WOC at high medical and social risk for preterm birth to gain an understanding of their pregnancy and birth experiences and to identify their unanswered questions and research priorities. Study methods and findings for the larger study are described in detail elsewhere [7, 30]. In the focus groups, WOC described the importance of their partner’s role during pregnancy. Among the study regions, Fresno has the highest incidence of preterm birth for large counties in California, with a reported 10.1% of live births [4]. Therefore, this city was chosen as the first site to host the MOC focus groups for the present study.

### Recruitment

The Fresno County Preterm Birth Initiative (PTBi-Fresno) and First Five Fresno County, supported by PTBi-CA served as the community-based organization (CBO) liaison for recruitment and host site, respectively. For the initial enrollment, male partners of women who participated in the WOC focus groups were contacted. Male participants were eligible if they self-identified as a POC, were 18 years or older, and could speak English. A recruitment flyer was created and distributed by PTBi-Fresno with the aim of enrolling 10 to 12 MOC. Additional MOC were recruited using snowball sampling, where current participants recruit additional participants for the study [31]. Snowball sampling was used after insufficient recruitment by flyer alone. Men were invited to two evening focus groups, two hours in duration each, that were held four weeks apart. Reminder and confirmation calls were made one week and two days before focus groups. The study was reviewed and certified exempt from human subjects protection procedures by the University of California, San Francisco Committee on Human Research (CHR) (#15–15,698). Participants received written information about the study, gave verbal consent to participate and signed informed consent for photography. Verbal consent was deemed sufficient by CHR as the study met the federal regulations in 45 CFR 46.104 (Common Rule, category 2) of a minimal risk research and therefore was exempt from federal policy for the protection of human subjects. Participants received $50 for participating in each session. Dinner and childcare were provided.

### Focus group procedures

Because all primary investigators were women, the CBO identified a male co-facilitator, who was also a PTBi-Fresno community leader, to co-facilitate groups. Research has shown that focus group facilitation may flow differently in groups that are mostly homogenous in regard to gender status [32]. Two of the primary investigators, and as well as the male facilitator, culturally identified as African American.

The focus groups followed the Research Priorities of Affected Communities (RPAC) protocol [30], with modifications by the research team and male co-facilitator to the facilitator guide to include additional questions to probe MOC experiences in accordance with the study specific aims. New questions asked about MOC personal health, support systems, and experience supporting their pregnant partner before, during and after birth (Table 1).

**Table 1:** Focus Group Questions

1) Describe your interactions with healthcare providers and staff	
2) What do you perceive as barriers and facilitators to seeking healthcare?	
3) What does health mean to you?	
4) What have you experienced while supporting your spouse or partner during pregnancy?	
5) How would you describe your own health during this time?	
6) What emotional and mental changes did you experience?	
7) How would you describe your support system?	
8) Were you working or taking care of other children?	
9) What have you observed in the experiences of other men of color in your family or community?	
10) Can you describe your experience(s) of seeking health care during your partner or spouses’ pregnancy?	
• What stands out in your mind? What questions did you have?	
• What would you change about this experience? What would you do differently?	
11) What things did you want to know that your health care provider couldn’t answer for you or your spouse?	
12) What things did you wonder about after talking with family or friends or after reading about them?	
13) What was your experience like with having a baby born early?	
14) When you hear that families of color in Fresno have such a high rates of preterm birth, what questions does that bring to mind for you?	
15) Do you have any unanswered questions or an uncertainty about what causes preterm birth and how to prevent it?	
16) What about treatment of babies and support for families?	
17) What questions do you have about the NICU experience?	
18) How do you think that answering that question through research will help other MOC/ families?	

In the first focus group, participants were asked to share their experiences of pregnancy and/or preterm birth. Experiences and perceptions were recorded on flip charts for participants and investigators to review together. Participants then continued to discuss their unanswered research questions as per the RPAC protocol. During the second focus group participants discussed their experiences and perceptions regarding men’s health and healthcare in greater detail, including their own interactions with HCPs, perceived barriers and facilitators to healthcare, and their definition of health. Additionally, participants were asked to share their reflections, as well as hopes for how this research could benefit their families and communities. The remainder of the second session focused on prioritizing their unanswered research questions per the RPAC protocol.

### Data collection and analysis

Focus groups were recorded, transcribed verbatim and edited to remove identifiers. Participants were notified beforehand of recorded portions of each meeting, and all identifying information was removed from transcripts to maintain anonymity. Session flip chart and researcher field notes provided additional information about participant responses and context. Notes were reviewed for content and merged on a single document for analysis. Thematic inductive analysis [33] was used to examine the data and report patterns and themes emerging from the data set. In order to become familiar with the data, recordings of focus groups were first listened to, while researchers simultaneously read over transcript approximately five times. Manual line-by-line coding was done by the first author to develop initial codes represented in the transcripts. Next, a second investigator (MM) conducted chunk-by-chunk coding after initial codes were identified. Initial codes and sample quotes were reviewed by the full research team to develop themes and sub-themes and a draft thematic map of men’s experiences was created by research team consensus, as suggested by Sandelowski and Barosso [34].

**Fig. 1:**
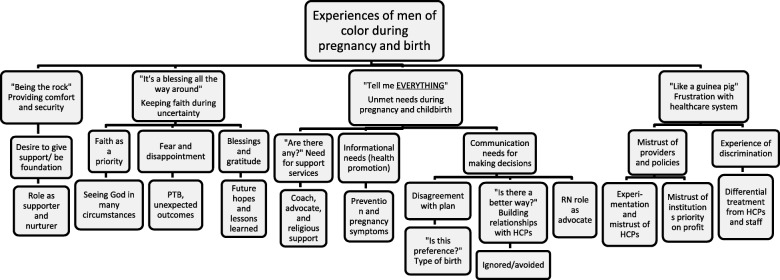
Thematic map of experiences of men of color during pregnancy and birth (*N* = 12). Abbreviations: PTB: preterm birth; RN: registered nurse; HCPs: healthcare providers

To ensure qualitative rigor and trustworthiness, concepts of credibility, transferability, dependability and confirmability were utilized [35]. Demographics of participants and geographic information was shared to describe the main characteristics of the population from which the sample was derived. Authors used introspection and discussed their own perspectives and biases that may arise during analysis. Additionally, a community report-back session was conducted to provide credibility through member checking to validate themes were a true reflection of experiences [35] . All of the original focus group participants as well as current members of the PTBi-Fresno Dad’s Council were invited. Eleven MOC participated, including five of the original focus group participants and the male co-facilitator. The details of the thematic analysis were discussed and the attendees agreed with identified themes and provided feedback regarding areas of emphasis and wording of the themes, sub-themes and selection of representative quotes. Participant feedback was incorporated into final analysis and thematic map (Fig. 1).

## Results

In total, twelve men participated in the focus groups (*N* = 12). Six men identified as Latino or Hispanic, five identified as African American, and one identified as Asian American. Eleven participants were married, ten were employed, and all twelve were from the Fresno County area. All participants had at least one child. Nine participants had partners who delivered one or more children by cesarean section. Seven participants had one or more children born preterm, with two participants having preterm twins. Two participants experienced neonatal death after a preterm birth.

### Men’s health

In order to contextualize the discussion around their experiences of pregnancy and birth, participants were asked general questions about their definition of health, barriers and facilitators to seeking care, and interactions with HCPs. Men defined good health to be a mix of healthy behaviors with physical, mental, spiritual, and relational components. Being healthy comprised more than staying active or having good nutrition. Health included maintaining close relationships with family, going to church or mass, coping well in stressful circumstances, and having an outlet for personal self-care. Barriers to seeking care included men’s expressed emotions of ambivalence and avoidance to seeking healthcare for themselves. For some, going to a healthcare provider *“costs too much, hurts too much, and takes too much time”.* Participants agreed with each other’s comments that is was easier to *“be your own doctor”*, delay treatment, and use natural or familial remedies for most health concerns. When asked about their own interactions with HCPs, there was very little discussion. The participants were often observed to redirect the conversation away from extended discussion of their own health and toward a focus on their wife’s pregnancy or health experiences of other family members.

### Experiences of pregnancy and birth

**Fig. 2:**
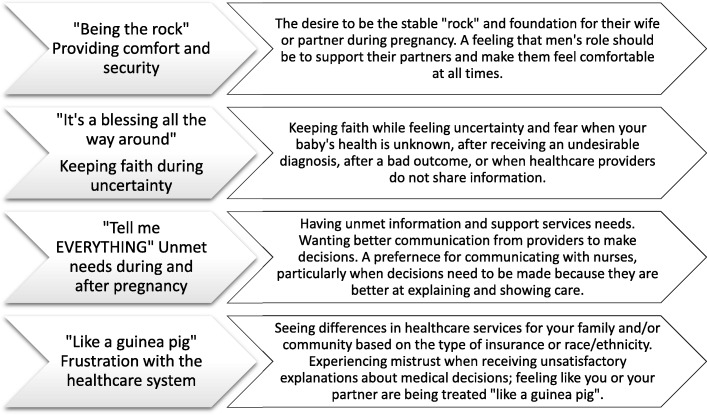
Four major themes of experiences of men of color during pregnancy and birth

Four major themes emerged from the analysis. The “*Being the Rock*” theme described men’s role in providing comfort and security for their pregnant spouse, their desire to give support, be the foundation, and serve as a supporter and nurturer for their family. The theme of “*It’s a blessing all the way around*” included subthemes of faith as a priority, fear and disappointment, and blessings and gratitude. The “*Tell me EVERYTHING*” theme encompasses unmet informational needs, support services and communication needs for making decisions during pregnancy and delivery. The final theme “*Like a guinea pig*” described frustration with the healthcare system, mistrust of providers and policies, and the experience of discrimination. The major themes and sub-themes are summarized in Fig. 2. Themes were experienced throughout antepartum, intrapartum, and postpartum periods, and are described below with specific illustrative quotes.

### “*Being the rock*” providing comfort and security

Every participant described the desire to be the stable “*rock*” and foundation for their wife or partner during pregnancy. Some men described this desire as innate and central to their role in the family. There was a general consensus that their role as fathers should be to support their partners and make them feel comfortable at all times. One participant described his experience of supporting his wife:

*If I want to cry, you know I’m not going to cry because I can’t allow her to feel that I’m weak, but she needs to have that rock... that’s kind of our role and that’s what we have to do, so we do it, but that can be difficult. (#5, Latino, preterm birth)*

Another participant explains his reaction when his wife wished she was pregnant again, so he could treat her differently:

*I didn’t realize I treated her any different, but she said I took care of her more and made sure I took care of a lot of things...I like to contribute to that, to the stress levels being lower. (#2, Latino, preterm birth)*

Participants agreed that their partner was a main priority, especially is it came closer to the time of birth. They took time off from work, made sure other children were cared for, and were available ‘on-call’ to meet the needs of their pregnant spouse. As the intensity of labor increased, or difficult decisions came up, participants felt their role as a stable support was crucial and continuous throughout this period.

### “*It’s a blessing all the way around*” keeping faith during uncertainty

A second central theme pertains to a balance between faith, fear, and uncertainty. Unprompted, many participants identified with a religious background, and some specifically with Christian or Catholic faith. Men expressed feeling uncertainty and fear, like a “roller-coaster”, when the status of their baby’s health was unknown, after receiving an undesirable diagnosis, after a bad outcome, or when HCPs did not have answers. Participants expressed that their faith in God kept them grounded when there was the threat of or actual experience of preterm birth or adverse birth outcomes. One participant explained his fear when his family found out their twins had twin-to-twin transfusion, which required surgery.

*Every day was scary... So we just kept strong in our faith...I’ll never forget because throughout the whole process there were so many people, complete strangers...and they would tell us like ‘I’m going to pray for you’... For me that was God in all those people. (#5, Latino, preterm birth)*

Three participants expressed fear and despondency after having a bad outcome or fetal death. One participant recalls:

*Every birth something really bad happens. He [the baby] kind of made us really afraid of having another baby. And my wife, she’s like ‘Just give it a try’. I wasn’t really feeling good about it, but it happened. (#3, African American, preterm birth)*

Participants who experienced a preterm birth generally appreciated all the support from NICU nurses and staff. One participant experienced multiple preterm births experiences, including fetal death. Processing through his experiences, he recalls his fears and gratitude.

*He was the smallest baby that they ever seen actually alive at that time because he wasn’t even a pound. I wouldn’t change any of it for the world because I feel like it made us all stronger...So yeah, it’s a blessing all the way around, you know? I learned so much with just NICU and preemies and the services that come behind that and the support groups... And we used every bit of it. (#4, African American, preterm birth)*

All participants had hopes for healthier babies for their Fresno communities and hoped research will find answers moving forward. They agreed that pregnancy *“should be the best time of your life”.*

### “*Tell me everything*” unmet needs during pregnancy and delivery

The majority of discussion for both focus groups was centered around the third theme of unmet needs of communication for themselves, to prevent complications, and make decisions.

“*Are there any?*” Needing support services for themselves and community: Participants experienced a lack of support services, had diverse informational needs, and desired better communication from providers to make decisions. Participants were often unaware of support services for themselves and their community. The ideal form of support described by participants came in the form of a “coach”, an advocate, or a pastor who would help make decisions and be a liaison between the hospital and family.

Support services were desired, not only for themselves, but for people in their community. Participants agreed that services are needed for their family members who do not know how to ask the right questions. Participants claimed that *“there’s nothing out there*” and these services *“should come without even asking”*. They inferred that this advocate would help families “*defend themselves*” and not be taken advantage of within the healthcare system.

Support was also desired from HCPs during moments of uncertainty. Participants acknowledged that providers cannot get too emotionally involved. However, they wanted providers to make more referrals, show emotion and be spiritual if appropriate. One participant recalled when a doctor *“broke the norm”*:

*I remember that day that my wife and the babies were having surgery, when the doctor told us, ‘They might not make it through the surgery’. I was in the waiting room and my knees were like shaking... Our doctor, the specialist, asked if he could pray with us. And to me that’s something I’ll never forget for a doctor to call you over, put his hands around you and your wife and pray. (# 5, Latino, preterm birth)*

Needing information to prevent complications and promote healthy pregnancy: Informational needs were also very important to participants. Participants wanted information about how to best prevent chronic conditions like diabetes or high blood pressure. They wanted to know if their own health had an influence of their child’s health, and why they experienced pregnancy symptoms like weight gain. Participants resorted to finding answers on Google and other internet sources when they did not get answers from their providers. One participant explained:

*I remember asking but I couldn’t get a clear-cut answer on if there was anything that we can do to prevent it [diabetes]. (#6, Latino, full term birth)*

Communication needs to make decisions: All participants expressed a strong desire for better communication between themselves and the providers. Multiple examples were shared of experiences where providers did not disclose or withheld desired information. Participants recognized a clear gap in communication and expressed that some providers were hard to understand. Additionally, participants wanted to make decisions for their wives and families, but do not feel supported to do so. Men wondered if there were standards for all providers to give the same type of communication to patients. They speculated that providers practice differently depending on where patients receive care. One participant asked:

*Does everybody receive the same standards? Because we know. We don’t want to say it. We know, but depending what hospital you go to, depending what insurance you might have, depending who you are, how you look, you’re not going to get those resources. (#5, Latino, preterm birth)*

After reflecting on previous pregnancies, one participant stated what he would tell his next provider:

*I had to tell my doctor, ‘Tell me everything. I don’t want to have to read between the lines.’ I want to know everything because I just do, you know. (#4, African American, preterm birth)*

Some participants gave recommendations for how providers should communicate. Participants agreed that information should be *given to all parents without asking*. This information was important for decision making and should be available to everyone. Participants hoped that in the future, all families will have the necessary information to make their own decisions.

“*Is there a better way?*” Desire to build relationships with HCPs: Participants felt that building a better relationship with their partner’s HCP was also a priority. Participants described feeling ignored and avoided during interactions while attending appointments with their wife.One participant recounted his experience while trying to seek information:

*Sometimes I would have questions and would try to talk to the nurse, and she would try to avoid like talking to me, for no reason you know. ... So she would try to have the conversation with my wife, not with me. Even though I was not the patient, but I wanted to know what was going on...I would see the monitor, that they put on my wife, with different lines. And I asked her [the nurse] ‘So what does those lines mean?’ I would see them going up and down. She didn’t say nothing. She didn’t explain it to me. I just wanted to know what it was, you know. I just wanted to hear an answer, ‘It was the heartbeat’. (#7, Latino, preterm birth)*

Preference of communication with nurses: Throughout the discussion, participants agreed they preferred communicating with nurses, and in particular during decision making. They felt nurses had the best relationship with their family and should have more power to make decisions. Participants found that nurses were an important source of support during a hospital stay. They trusted nurses more because they had time to develop a relationship. Participants questioned the significance of the HCP role and advocated for increased nurse authority.

One participant said:

*And I always found myself, ‘Okay, doctor. You can go ahead and leave. I’d rather just talk to the nurse...Do nurses have the authority to make decisions? They get to know the baby. They’re coming in every hour or so to check on the baby. (#2, Latino, preterm birth)*

“*Is this preference or what is best for baby?*” Disagreement with type of birth: Participants were generally frustrated and confused with the type of birth their wife or partner experienced. Nine of the twelve men experienced unexpected C-sections and still had many doubts that the right decision was made for their family. Some participants valued natural childbirth and perceived C-sections to be used only in emergencies. Some did their own investigation online and compared their experience to other places and even countries. One participant said:

*But I just didn’t understand why, the reason why they were enforcing the C-section on her if she wanted to have a vaginal birth. But I mean they told her more than three times. And my wife, she looked up on Google that I think in Japan they promote vaginal birth. They don’t use C-sections unless it is very very necessary, but otherwise they won’t do C-sections. (#8, Asian, full term birth)*

### “*Like a guinea pig*” frustration with the healthcare system

Experience of discrimination based on health insurance: Throughout the conversation, participants described a difference in receiving healthcare services for their family and/or community due to the type of insurance they held. This difference was seen in the treatment of staff and providers, wait times at clinics, and services received.One participant explained the difference he experienced:

*I’ve noticed, my daughters have Medi-Cal and I have regular insurance. I can see the difference. The way they treat me for sure. When I go to my doctor, which is you know just a regular HMO, they are a little nicer, curious. Medi-Cal, when I take my daughters, they are short, and ... rough. And I just kind of treat them nice and then they realize, “Oh Okay, he’s okay”. I can tell the difference, like immediately... It starts at the front desk. (#2, Latino, preterm birth)*

Another participant said:

*Unfortunately, that whole insurance thing is real, man. I worked at a hospital and unfortunately people that had Medi-Cal, like that need certain scans, x-rays, or whatever, it shows what insurance you have right there. And if you have Medi-Cal you are going to the back of the bus. (#4, African American, preterm birth)*

Experimentation and mistrust of providers: Medical mistrust was a final sub-theme that emerged as participants tried to find explanations for decisions being made about their wives’ pregnancy and/or delivery. During the antenatal period, certain tests and procedures seemed experimental and without reason. Some felt as if they were being used for research purposes.One participant shared his experience when his wife had diabetes:

*Well, why is it she [wife] took all those notes down and she would keep track of everything, and then in an instant they made a decision? [C-section] Why does that happen? What's the purpose of all that detail and coming to all those appointments? What's the purpose of all her work and effort when it looks like they didn't even use it? (#1, Latino, preterm birth)*

A participant answered:

*Like a guinea pig almost, like use us for find the results, you know? You’re thinking, okay, this is going to fix the problem. But really they're still trying to figure out the problem, but using you. (#9, African American, preterm birth)*

Another stated:

*Is it legal for them to use you, almost as research, without you knowing basically? (#1, Latino, preterm birth)*

Mistrust of institutions: Perceived priority on making profit: Participants believed there was a priority of institutions and hospitals on making a profit, which may negatively impact families. Fathers were suspicious of research findings, hospital visits, and cesarean section rates. Participants also wanted to understand if there were more Medi-Cal patients who had cesarean sections. One participant asked:

*You [hospitals] have to have so many C-sections to make sure you make, you turn a profit....Is it a quota you have to meet? (#10, African American, full term birth)*

Another participant added:

*But with cesarean sections, you know, you get cut. You’ve got to go back to make sure you heal up. They get paid for every time you visit, you know. (#9, African American, preterm birth)*

## Discussion

This study is one of the first detailed explorations of the pregnancy and birth experiences of MOC in a community at high risk for preterm birth and adverse birth outcomes. Study findings revealed MOC wanted to have an active role in their partner’s pregnancy and birth and to serve as an advocate and ally to their partner and child. Many of the participants cited the importance of their faith in God in helping them cope with adversity and uncertainty of a difficult pregnancy or birth, and they also described experiences of discrimination, and mistrust of HCP and the healthcare system.

The dominant narrative from previous research has identified themes of role transition, mixed emotions, neglect, and unmet informational needs in fathers’ experience of pregnancy and childbirth [8–14]. Specific to the experience of preterm birth, a metasynthesis of 24 qualitative studies of the experiences of fathers of preterm infants receiving care in the NICU identified common themes of proximity, parental autonomy, vulnerability, communication, and exclusion and isolation and highlighted the powerful role of NICU staff in creating opportunities or barriers to fathers’ involvement in their infant’s caregiving [36]. Another recent systemic review of 15 quantitative observational studies found that fathers of infants in NICUs experienced higher levels of stress than fathers of healthy infants, related to parental role alterations, infant appearance, environment and staff communication [37].

Men of color in this study experienced similar positive emotions during their partners’ pregnancies and a strong sense of identity in supporting and caring for their wives and partners, describing their role as “Being the Rock” and supporting and nurturing their partners and family. However, they also experienced neglect from prenatal and perinatal healthcare providers and lacked information, as found by others [12–14]. Men of color who had preterm children in this study, described feelings of stress, as if they were on a “roller coaster” ride, unmet information needs, lack of support services and communication challenges.

This research also brings to light new themes specific to MOC, which have not been described in previous research with predominately White men. New themes described participant’s desire for external support in the form of an advocate or coach to help “*defend themselves*” in a healthcare system that did not recognize their information and communication needs. Most participants coped by depending on faith and prayer during uncertainty. For MOC, unmet informational needs were often compounded by mistrust. Fathers mistrusted providers when they were not given clear rationale or explanations for management decisions. They advocated for nurses to have more authority for decision-making because of nurses’ investment in patient and family relationships during healthcare encounters. Overwhelmingly, men reported they did not have enough support to make decisions for their family and felt providers were withholding information. Participants raised deep concerns and were troubled about the differential treatment of their families and community who had public insurance. Notably, they valued the natural process of birth and wondered if their wives received cesarean sections because it brought profit to the hospital. The MOC in this study also expressed concern about the high rates of preterm birth in their community and expressed hoped for a brighter future. The current narrative about fatherhood in society has not sufficiently addressed these new themes for men of any ethnicity, and further research is needed.

Further research is also needed to explore the experience of MOC whose partners are at higher risk specifically for preterm birth. Many questions still remain unanswered in the literature. For example: Do MOC living in high income communities have different experiences than those living in low income communities? Moreover, there is no research on whether MOC have different experiences if a midwife or a physician attends their partner’s birth. Additionally, further research is needed on the types of support that MOC desire. Would MOC have similar needs if they had a doula present? Do MOC in the NICU also feel supported by HCPs and encouraged to participate in caregiving? These are just a few of the many unanswered research questions that should be pursued as part of a major effort to address this critical knowledge gap and inform interventions to improve preterm birth outcomes.

As further research is published and the dominant narrative shifts and begins to include experience of MOC, midwives and other HCP and health system leaders can gain insights to better serve MOC and their families. Healthcare providers play a major role in the experience of patients and their families and can help mitigate prior negative experiences and reinforce positives ones. Based on the experience of MOC in this study, the following recommendations for HCPs are made: 1) Expect that MOC may define their role as being “The Rock” and provider for their families and, if confirmed, work with them to identify ways to support them in this role; 2) Assess the need for increased services in the form of religious support, coach or advocate, so that MOC can fully participate in their partner’s pregnancy and birth and fulfill their desired roles; 3) Implement strategies to improve patient/family-provider communication, such as meaningfully including MOC in discussions during the antenatal and postnatal periods in alignment with the pregnant partner’s preferences and values; 4) Do not withhold information - give full explanations and rationales for recommending management plans and be honest if the answer is unknown; 5) Validate the importance of the nurse-patient/family relationship and acknowledge nurses as a key participant in patient care; and 6) Ask pregnant partners and MOC about their own experiences with healthcare. If they describe previous trauma, discrimination, or lack of trust, explain how those situations will be prevented/addressed during the current pregnancy and birth care. This last recommendation is particularly important as racism is a common experience of most POC [38]. By acknowledging this common experience, trust can be built and enable MOC to feel more comfortable, communicate more openly and become allies in improving preterm birth outcomes for their families and for their wider communities.

Healthcare system-level interventions are needed to address the issues identified by MOC in this study. Recent literature stresses the importance of interventions that involve men in maternal health and calls for health systems to engage men as active participants in health promotion strategy [38]. First, it is imperative to involve MOC by asking them what ideas they have to better integrate men into maternity and newborn care. Second, explicitly acknowledging and addressing the lack of diversity within midwifery, nursing and medical professions may help decrease provider mistrust and miscommunication. Third, hospital administrations and institutions can provide accessible information about profit gains for cesareans, tests, and procedures during delivery. Building better relationships and accountability between hospitals and their patient populations can limit institution mistrust, especially for Black families who are at greatest risk for preterm birth [3] and have been historically and presently abused within the U.S. healthcare system [39–42].

### Limitations and strengths

These study findings should be considered in light of the limitations and strengths of the study. First, the findings are based on the experiences of a small sample of MOC from Fresno, CA; therefore, they may not reflect experiences of the larger MOC population. Second, we did not collect data on the length of time between the focus groups and the children’s births. However, fathers’ feelings about their experiences of pregnancy and childbirth may change over time and should be explored in future research. Third, although the sample was a racially and ethnically mixed group, and common themes emerged, further research with groups that are racially/ethnically homogenous may reveal additional insights. Strengths of this research included the rigorous review of prior literature in developing the novel research question, the CBO partnership in developing the protocol and session facilitation, and the rigorous confirmation of the results with participants and non-participant MOC from the same community. Additional qualitative and quatitative research, including surveys, focus groups and interviews, are needed to further expand and confirm the findings from this exploratory study.

## Conclusions

This is, to our knowledge, the first qualitative study to describe the unique experiences of MOC during their partner’s pregnancy, birth and their child’s newborn care. The major thematic findings that MOC view their main role as supporting their partner during pregnancy and birth, their focus on faith during uncertainty, their own unmet information and support needs, and frustrations with the healthcare system, can guide future research as well as clinical and institutional practice improvements. Larger studies are needed to assess the impact of pregnancy and postnatal care provision on MOC in the U.S. Interventions to develop father-centered communication is needed to help rebuild trust for MOC at all phases of maternal and neonatal care. HCPs also need to acknowledge that MOC are likely to have experienced discrimination and mistrust in healthcare encounters and assess needs for support and involvement in decision- making. As WOC are at greater social and medical risk for preterm birth, they and their male partners should be directly included in all future maternal-newborn research and health service redesign.

## Data Availability

All data generated or analyzed during this study are included in this published article [and its supplementary information files]. Study data, methods and training video available from: https://pretermbirthca.ucsf.edu/developing-research-strategy-partnership-communities-affected-preterm-birth.

## Abbreviations

CBO: community-based organization
HCPs: healthcare providers
MOC: men of color
POC: people of color
PTBi: Preterm Birth Initiative
U.S: United States
WOC: women of color
